# Biosafety Level 4 Laboratory User Training Program, China

**DOI:** 10.3201/eid2505.180220

**Published:** 2019-05

**Authors:** Han Xia, Yi Huang, Haixia Ma, Bobo Liu, Weiwei Xie, Donglin Song, Zhiming Yuan

**Affiliations:** Wuhan Institute of Virology, Chinese Academy of Sciences, Wuhan, China

**Keywords:** biosafety training, BSL-4, China, high-containment laboratory, laboratory, pathogens, personnel certification, high-consequence pathogens

## Abstract

Experienced, qualified personnel certified to work in high-level biocontainment laboratories contribute to the safe operation of these facilities. China began a training program for laboratory users after establishing its first Biosafety Level 4 laboratory, the Wuhan National Biosafety Laboratory (Level 4) of the Chinese Academy of Sciences. We provide an overview of the content of this training program, which can serve as a reference for developing national norms for high-containment laboratory training.

Over the last 2 decades, numerous infectious diseases, including Ebola, Marburg hemorrhagic fever, Crimean-Congo hemorrhagic fever, Lassa fever, severe acute respiratory syndrome (SARS), avian influenza A(H5N1), Rift Valley fever, and Nipah and Hendra viral diseases, have emerged and re-emerged. These infectious diseases pose serious threats to public health and global economies. For example, 28,616 suspected cases and 11,310 deaths were reported during the large and complex Ebola outbreak in West Africa during 2013–2016 (*1*). In addition, as the threat of terrorism increases globally, the risk for bioterrorism is expected to increase as well. To prepare for biological threats, scientists must research dangerous pathogens to develop effective methods to prevent, diagnose, and treat the diseases caused by them. 

Laboratories are classified into biocontainment levels 1–4, depending on the pathogenicity of microbes investigated. Biosafety Level 4 (BSL-4) laboratories investigate the most dangerous pathogens and have the maximum biocontainment levels. Microbes contained in BSL-4 laboratories pose a significant risk for transmission and are frequently fatal; most have no reliable cure. BSL-4 laboratories provide a safe environment in which laboratory staff can work with and study these highly pathogenic microbes. 

After the 2003 SARS epidemic, the government of China initiated a plan to construct a national high-level biosafety laboratory system to prepare for and respond to future infectious disease outbreaks. One of the goals was to build a BSL-4 laboratory that meets the national and international standards for diagnosing, researching, and developing antiviral drugs and vaccines while additionally preserving highly pathogenic BSL-4 agents for future scientific research. Within the framework of the Sino-French Cooperation Agreement on Emerging Infectious Diseases Prevention and Control (*2*) signed in October 2004, China constructed its first BSL-4 laboratory, the Wuhan National Biosafety Laboratory (Level 4) of the Chinese Academy of Sciences, in 2015. During construction, prospective BSL-4 laboratory staff members visited France, the United States, or Australia for BSL-4 training and capacity building. After 2 years of testing and commissioning, Wuhan BSL-4 laboratory passed a series of assessments, and the China National Accreditation Service for Conformity Assessment certified it as meeting the highest biosafety standard in January 2017 (*3*). In August 2017, the National Health Commission of China approved research activities involving Ebola, Nipah, and Crimean-Congo hemorrhagic fever viruses at the Wuhan BSL-4 laboratory (*4*).

The safety and function of a BSL-4 laboratory rely not only on the containment facility and biosafety management systems but also on highly qualified and experienced staff. Many recorded laboratory accidents are related to personnel error. Qualified staff ensure the efficacy and safety of a high-containment laboratory. Developing an appropriate and rigorous BSL-4 laboratory user training system is an essential prerequisite for ensuring the safety of laboratory workers, operations, and the environment (*5*,*6*). We designed a training program for BSL-4 laboratory users that follows local, national, and international guidelines and regulations. Our program ensures the safety and security of staff involved in research at the Wuhan BSL-4 laboratory. 

## Training Program Overview

We developed our BSL-4 laboratory user training program in 2017 to provide continual on-the-job training. The main goal of the program is to prepare laboratorians to work at our maximum biocontainment laboratory. Before being admitted to the training program, applicants must demonstrate proficient work experience in lower containment level laboratories, particularly BSL-3, and undergo medical examinations and security checks. We constructed a realistic training laboratory with bioseal doors, pressure sensors, a chemical shower room, air connection ports, biosafety cabinets (BSCs), an autoclave, and general laboratory equipment (Figure, panel A). In this laboratory, trainees gain experience wearing a positive-pressure suit (Figure, panel B) and become familiar with working in the BSL-4 environment without actually handling microbes. To ensure documentation of all training, we developed online training management software, which supports registration, staff pre-assessment, needs assessments, a theoretical examination, record keeping, and analysis of training efficiency.

**Figure:**
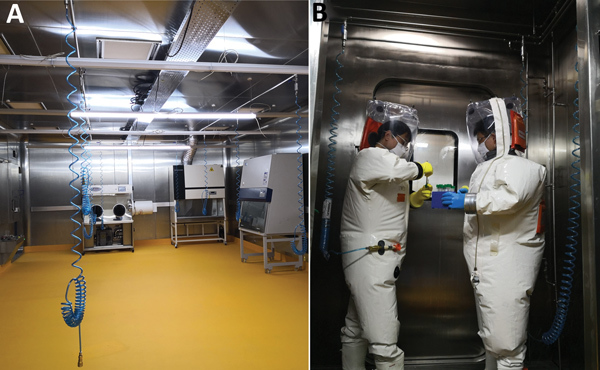
Photographs of the Biosafety Level 4 (BSL-4) training laboratory for China’s Wuhan National Biosafety Laboratory (Level 4) of the Chinese Academy of Sciences. The training laboratory is designed to simulate working conditions in a BSL-4 laboratory. A) Inside the BSL-4 training laboratory, where trainees safely learn to use necessary equipment such as air connections ports and biosafety cabinets. B) Trainees wearing positive pressure suits practice skills in the BSL-4 training laboratory.

The training team is composed of existing trained staff or outsourced qualified mentors. Currently, we include 3 categories of trainees according to their duties: laboratory managers or biosafety professionals, operations or maintenance staff members, and researchers or technicians who work with infectious agents in vitro or in vivo.

## Training Process

Our BSL-4 users training occurs in 3 phases: a pre-assessment, theoretical and practicum training, and a final assessment (Appendix). Training comprises small group lectures, hands-on practice in the training laboratory, and mentored work in the functioning BSL-4 laboratory. Depending on their role in the laboratory, trainees complete required and elective modules and a specified number of mentored hours and entries into the laboratory to receive certification. In addition, we provide supplemental training after any procedural or policy changes and annual refresher training for experienced workers. 

### Pre-Assessment

The pre-assessment helps us determine trainees’ experience and identify their training needs. During pre-assessment, trainees provide information on group 1–4 pathogens they have previously handled, their experience in biosafety settings, animal species with which they have working experience, techniques with which they have experience, whether they are taking training for the first time or as a refresher, which training level they are requesting, and agents they will handle and their responsibilities in the laboratory after training. The trainee also takes an examination targeting their user category, manger, operations and maintenance, or researcher. This examination provides guidelines for topics or content that the trainee should focus on during the theoretical training.

### Theoretical Training

The theoretical courses are designed to help trainees gain a comprehensive understanding of biosafety and biosecurity principles and regulations. Theoretical training provides an overview of the features of the maximum biocontainment laboratory, especially the design features, maintenance and management, the code of practice, the key facilities and equipment, and the standard operating procedures for working at our BSL-4 laboratory.

The classroom-based theoretical coursework consists of 14 topics with ≈45 minutes of lectures and discussions per topic (Appendix). The trainees complete courses on different topics according to their job category. After completing all required courses in the theoretical series, trainees must pass an examination with a score >90% before proceeding to the hands-on courses in our training laboratory. Trainees who do not pass receive further instruction and review topics they did not pass before retaking the test.

### Hands-On Training

The hands-on practicum provides training in a laboratory setting, strengthens trainees’ knowledge and skills, and delivers a comprehensive orientation to our BSL-4 facility. During hands-on training, staff become familiar with laboratory features, such as airtight doors, dedicated airflow supply and exhaust systems, autoclave, chemical shower, negative-pressure environment, personal protective equipment, standard operating procedures, and safety procedures, including alarms and emergency operations.

Hands-on training includes 6 topics (Appendix) and is conducted through demonstration and practice using non-infectious materials at our training laboratory. Trainees take topics according to their job category. During these sessions, an assigned mentor or trainer demonstrates the correct procedures before the trainee practices under observation. In this phase, all trainees learn about the positive pressure suit, including how to inspect and wear the suit and use compressed air hoses. In addition, they learn emergency plans and proper techniques for entering and exiting the BSL-4 laboratory, working in or using laboratory equipment, disinfecting surfaces, disposing of waste, cleaning spills, removing equipment or material from the laboratory, and conducting decontamination. Because laboratory accidents often involve animal bites and sharps, we require an additional module specific to these issues for staff who will work with animals; those whose work is mainly in vivo may take this course as an elective. 

On completion of this section, the mentor or trainer observes the trainee performing assigned procedures and evaluates whether they are performed correctly per standard operating procedures. These evaluations are recorded through our online training management software tool.

### Mentored On-the-Job Training

Before they can be certified, trainees who will work at the BSL-4 laboratory must complete a specified number of hours and entries into the laboratory in mentored on-the-job training (Appendix). During this phase, trainees work in a functioning BSL-4 laboratory under supervision of a senior staff scientist or other experienced laboratorians. After an orientation, they perform specific tasks, such as routine inspection and BSC testing, moving equipment, setting up BSCs before conducting experiments, disinfecting surfaces and removing generated waste, centrifugation, virus propagation and storage, plaque assays, inoculation, and animal care and use. 

The assigned mentor evaluates the trainee’s performance, advises on safe and secure operations, and records areas in which the trainee needs further instruction or practice. The mentor then makes recommendations on whether the trainee is prepared for independent access to laboratory facilities.

### Final Assessment and Certification

After a trainee completes hands-on skills assessments and passes the final theoretical exam, the laboratory director reviews training records, laboratory work hours and entries, and the mentors’ recommendations before authorizing a certification. Depending on the level of work experience and job categories, staff are certified at 3 grades: Grade Green personnel must be under the guidance of internal or external experts or mentors when accessing the BSL-4 facility; Grade Orange personnel have independent access to the BSL-4 facility; and Grade Red personnel have independent access directly to the BSL-4 facility and are qualified to be mentors and instructors (Appendix).

## Discussion

According to China’s “One Belt, One Road” initiative, the chance that exotic pathogens could be brought into the country has dramatically increased (*7*). Our new BSL-4 facility will play an integral role in preventing and controlling highly pathogenic microbes. To safely operate this facility, we designed a training program that ensures all personnel meet the institutional, national, and international standards for working in maximum-containment laboratories.

In preparation for the opening of the Wuhan BSL-4, we engaged in short- and long-term personnel exchanges focused on biosafety training through international cooperation (*8*). Four staff members visited the P4 Jean Mérieux-Inserm Laboratory in Lyon, France; 2 visited Galveston National Laboratory, The University of Texas Medical Branch at Galveston, Texas, USA; and 1 visited the Australian Animal Health Laboratory, Geelong, Victoria, Australia for training and certification on BSL-4 laboratory operations, maintenance, and scientific or support work. These members are now the main instructors for our BSL-4 laboratory user training program. 

Rather than being standardized, our training is specialized to fundamentally cover different BSL-4 users, including administrators and management, biosafety professionals, operations and maintenance staff, and researchers and technicians who currently work in the laboratory. The theoretical coursework is designed to help trainees understand the features of the BSL-4 laboratory and prepares them to enter the laboratory environment. We constructed the first BSL-4 training laboratory in China with the sole purpose of providing hands-on practicum for staff. This laboratory gives staff a safe environment in which they can learn all routine and emergency procedures of high-containment laboratories without the risk of exposure to dangerous pathogens. In addition, we developed an online training management software tool to support the training program and track participants’ progress towards certification.

We plan to incorporate additional user training, such as training for temporary or visiting workers from outside the institution who currently do not have access to our laboratory. In addition, we are planning specific training designed for emergency first responders, such as security staff at the institute and the city’s police and fire departments. Because these groups are tasked with responding to incidents, such as terrorism or fires, they need to be familiar with the complex design and mechanical and engineering features of the BSL-4 facility. Our expanded training will orient them to the laboratory and its operating systems so they can respond as safely as possible to any emergency at our facility.

Our rigorous training program will reduce the risk of harm or exposure to laboratory staff working with highly pathogenic agents. We encouraged all laboratory users to provide feedback and thoughts regarding how to improve and further advance our training program. China intends to build 5–7 high-containment laboratories by 2025 (*9*). Our BSL-4 laboratory worker training system is the starting point for developing national norms for high-containment laboratory training and preparing qualified, maximum biocontainment laboratory scientists and facility operations specialists.

Appendix Overview of the Wuhan National Biosafety Laboratory (Level 4) User Training Program.

## References

[1] World Health Organization. Ebola data and statistics. 2016 May 11 [cited 2018 Feb 5]. http://apps.who.int/gho/data/node.ebola-sitrep

[2] Ministry of Foreign Affairs of People’s Republic of China. The bilateral relations between the People's Republic of China and the Republic of France [in Chinese]. 2019 Jan 1 [cited 2019 Mar 11]. https://www.fmprc.gov.cn/web/gjhdq_676201/gj_676203/oz_678770/1206_679134/sbgx_679138

[3] Cyranoski D. Inside the Chinese lab poised to study world’s most dangerous pathogens. Nature. 2017;542:399–400. 10.1038/nature.2017.21487 28230144

[4] National Health Commission of People’s Republic of China. China’s first bio-safety level 4 lab put into operation [in Chinese]. 2018 Jan 1 [cited 2018 Dec 5]. http://www.nhc.gov.cn/zhuz/xwfb/201801/90502af790884777b2844ad7dc8c4c2f.shtml

[5] Le Duc JW, Anderson K, Bloom ME, Estep JE, Feldmann H, Geisbert JB, et al. Framework for leadership and training of Biosafety Level 4 laboratory workers. Emerg Infect Dis. 2008;14:1685–8. 10.3201/eid1411.080741 18976549 PMC2630756

[6] Nisii C, Castilletti C, Raoul H, Hewson R, Brown D, Gopal R, et al. Biosafety Level-4 laboratories in Europe: opportunities for public health, diagnostics, and research. PLoS Pathog. 2013;9:e1003105. 10.1371/journal.ppat.1003105 23349630 PMC3547859

[7] National Development and Reform Commission of People’s Republic of China. Vision and actions on jointly building Silk Road economic belt and 21st-Century maritime Silk Road [in Chinese]. 2015 March 30 [cited 2018 Dec 5]. http://www.ndrc.gov.cn/gzdt/201503/t20150330_669162.html

[8] Le Duc JW, Yuan Z. Network for safe and secure labs. Science. 2018;362:267. 10.1126/science.aav7120 30337385

[9] National Development and Reform Commission of People’s Republic of China. The planning (year 2016–2025) for the construction of high-level biosafety laboratory system [in Chinese]. 2016 Nov 30 [cited 2018 Dec 5]. http://www.ndrc.gov.cn/zcfb/zcfbtz/201612/t20161220_830455.html

